# The epidemiology of moral bioenhancement

**DOI:** 10.1007/s11019-020-09980-1

**Published:** 2020-10-06

**Authors:** R. B. Gibson

**Affiliations:** Centre for Social Ethics and Policy, The Law School, The University of Manchester, Williamson Building, 176 Oxford Road, Manchester, M13 9QQ UK

**Keywords:** Moral bioenhancement, Epidemiology, Social contagions, Bioethics

## Abstract

In their 2008 paper, Persson and Savulescu suggest that for moral bioenhancement (MBE) to be effective at eliminating the danger of ‘ultimate harm’ the intervention would need to be compulsory. This is because those most in need of MBE would be least likely to undergo the intervention voluntarily. By drawing on concepts and theories from epidemiology, this paper will suggest that MBE may not need to be universal and compulsory to be effective at significantly improving the collective moral standing of a human populace and reducing the threat of ultimate harm. It will identify similarities between the mechanisms that allow biological contagions (such as a virus) and behaviours (such as those concerned with ethical and unethical actions) to develop, spread, and be reinforced within a population. It will then go onto suggest that, just as with the epidemiological principle of herd immunity, if enough people underwent MBE to reach a minimum threshold then the incidence and spread of immoral behaviours could be significantly reduced, even in those who have not received MBE.

## Introduction

In their 2008 paper, Persson and Savulescu explored whether, given the increasingly destructive potential posed by scientific and technological advancements such as nuclear and biological warfare, moral bioenhancement (MBE) should be developed as a means of closing the gap between our moral faculties and destructive capacities (Persson and Savulescu 2008). They argued that if the development of our destructive capabilities, fuelled by exponential increases in our cognitive faculties, continues unabated then it will need to be accompanied by research into, and the application of, MBE. This is necessary, they argue, as a means to avoid what they term ‘ultimate harm’ (UH); humanity’s self-inflicted destruction. They posit that because those who are most likely to bring about this UH are those same individuals who are least likely to undergo voluntary MBE if developed and proven to be safe, the intervention should be applied on a universal and compulsory scale. Thus, to ensure the efficacy of MBE, it should be made obligatory, regardless of a person’s original moral baseline.

While they defend this view in their 2013 paper (Persson and Savulescu 2013), their more recent work has moved away from this extreme position (Persson and Savulescu 2015, 2019a, b). However, the question of whether MBE should be compulsory, specifically to be effective at reducing or eliminating the risk of UH, has remained relatively unexamined. This paper will address this gap in the literature. To do this, it will explore the idea that by understanding behaviour according to epidemiological models, and specifically in reference to the phenomena of herd immunity, it may be possible for MBE to be effective at reducing or altogether avoiding the risk of UH without a need for a compulsory enhancement programme.^1^

First, this paper will provide a fuller account of the erroneous reasons why, according to Persson and Savulescu, MBE would need to be compulsory. Second, a justification for the use of epidemiological models as a means of understanding data transfer and the phenomena of ‘transmission’ outside the health sciences will be provided, drawing on historical and contemporary examples of its application in a wide range of fields. Third, a brief account of how the vaccine epidemiological effect of herd immunity works will be provided with specific attention being drawn to herd immunity thresholds and infection chain disruption. Fourth, this paper will argue that there are similarities between the mechanisms that allow biological contagions (such as a virus) and behaviours (such as those concerned with ethical and unethical actions) to spread and be reinforced within a population. Finally, this paper will argue that if MBE was made voluntarily available, it could have the desired effect of managing the risk of UH provided that enough of the population took the enhancement to reach the behavioural equivalent of the herd immunity threshold. However, before continuing onto the main body of this paper, some scene-setting is required.

## Assumptions underlying the compulsory enhancement question

Over the past decade, there has been considerable ethical debate about the conception, development, and theoretical implementation of MBE. As identified by Specker et al. (2014), these debates can be grouped into the following six categories: (1) the need for MBE; (2) the ability to reach a consensus on what MBE should involve or what effect it should have; (3) how feasible MBE is in relation to the current status of scientific research; (4) the ethical value of the means and processes at which we arrive at an improved morality; (5) what impact such MBE would have on freedom, identity, and autonomy on an individual level; and (6) what effects MBE would have on social and group dynamics. To focus on the question of whether MBE should be employed on a universal compulsory level, this paper makes several assumptions about some of the issues in the broader MBE debate.^2^

First, the question this paper is seeking to answer is situated on the assumption that human moral behaviour, agency, insight, and willpower, require improvement and that for this reason, MBE is needed. Few would argue that there is no such need for improvement. Crucially, however, while ‘traditional’ moral enhancement is necessary and can make some progress towards addressing human moral fault,^3^ it is not up to the task of addressing our collective moral deficiency on its own.^4^

Second, it will be assumed that there is no principled difference between MBE and ‘traditional’ moral enhancement. Neither is seen as being superior to the other regarding ethical permissibility or desirability. Thus, there is no innate harm in pursuing MBE when compared to ‘traditional’ enhancement techniques.

Third, this paper will assume that a consensus has been reached regarding what constitutes the ethical good and what is morally desirable. Due to the pluralistic milieu in which we inhabit, there is considerable debate surrounding what traits, behaviours, and positions are considered socially and ethically desirable. To advance the debate about MBE provision, therefore, it is assumed that such desirable characteristics have been identified and agreed.

Forth, while proponents have been optimistic about current and future scientific research (Persson and Savulescu 2012; Douglas 2008; Degrazia 2014), opponents claim that due to the complexity of our psychology and biology it is unlikely that we will be able to create any form of effective MBE (Specker et al. 2014; Arnhart 2010; Sparrow 2014; Wiseman 2016; Kudlek 2019). This paper assumes that a method of MBE has been developed which demonstrably makes individuals more inclined to act in a way that has been decided on as morally desirable.

Finally, some assumptions must be made regarding individual free will and autonomy, specifically whether those individuals who undergo MBE would still be able to make independent and autonomous decisions. This paper will assume that the form of MBE that could/would be provided is one which does not impact on the capacity for autonomous decision making. Those who take MBE are as free to make their own decisions without undue influence as they were before taking the enhancement. It is just that they are likely to base those decisions on an ‘improved value system’.

Following all of these assumptions, we can identify the characteristics of the MBE which will be under discussion; namely, a form of MBE which has been shown to promote universally ethically desirable behaviours, under both empirical tests and in ‘real world’ situations, without any impact on cognitive ability or autonomous decision making. While this may not necessarily be the form of MBE that comes into existence, if any do at all, a MBE with these qualities would arguably be the most desirable and least contentious form of the intervention.

## The compulsory MBE debate so far

Persson and Savulescu presented the argument that for humanity to have a chance of preventing potentially world-ending events, the gap between our collective cognitive abilities and our collective moral capabilities must be reduced.^5^ Instrumental in achieving this reduction is an effective form of MBE. This intervention would be developed and implemented to enhance our moral capacity beyond its current point, and by doing so, this would enable us to employ a morality better suited to ‘good’ uses of our cognitive abilities.

They argue that such moral enhancements are required as a result of our achievements in collective cognitive enhancement, which have enabled us, as a species, to develop to the level of scientific and technological success at which we currently sit. This success has allowed for the development of several avenues through which UH can be brought into realisation. Some of these have been created to be deliberately destructive (nuclear weapons, biological warfare, the proliferation of global injustices via mass-misinformation). In contrast, others have a foundation in a prevailing apathy to alter behaviour in order to avoid them (climate change, antibiotic resistance, the spread of zoonotic diseases). Consequentially, “[t]he expansion of our powers of action as the result of technological progress must be balanced by moral enhancement on our part” (Savulescu and Persson 2012, p. 400). A failure to do so would allow for a collective cognitive capacity that eclipses the collective moral capacity, resulting in deliberative or accidental UH.

Additionally, Persson and Savulescu argue that as the capabilities of science and technology increase, so too does the likelihood that small groups of people, or even individuals, will be able to procure weapons or technologies enabling them to cause significant harm to millions of people (Persson and Savulescu 2008, p. 168). Furthermore, even if the number of individuals who would wish to use these weapons to such effect is only a tiny fraction of human beings, due to the sheer numbers of people alive today, this fraction would be large enough to present a credible threat. Persson and Savulescu argue, therefore, that the progress of science is potentially making the world a worse place in which to exist due to the increasing likelihood of both destructive climate change or the misuse/mismanagement or unjust deployment of weapons of mass destruction. They conclude that:

If safe moral enhancements are ever developed, there are strong reasons to believe that their use should be obligatory, like education or fluoride in the water, since those who should take them are least likely to be inclined to use them. *That is, safe, effective moral enhancement would be compulsory* (Persson and Savulescu 2008, p. 174).

For Persson and Savulescu then, *compulsory* MBE is necessary for the intervention to be effective at its goal of preventing UH. Affording individuals the choice regarding whether they should undergo MBE allows the risk of UH to remain a distinct threat as those most in need of enhancement are those least likely to undergo it. Given that developments in science and technology make it increasingly more accessible for smaller and smaller groups of people to bring about this destruction, leaving these individuals unenhanced is too great a risk to take.

Persson and Savulescu’s conclusion that MBE would need to be compulsory has, unsurprisingly, met several counterarguments. Harris offers a nuanced critique of specific forms of MBE that enhance morality through the direct alteration of emotions via biomedical means. This contrasts his tempered enthusiasm for moral enhancement via the alteration of cognitive functions (Harris 2011, 2013, 2014, 2016). Harris’ trepidation regarding specific forms of MBE is expressed chiefly in three main arguments.

Firstly, he is sceptical of the necessity of such interventions as a single measure of combatting unethical behaviour, believing that rather than an emotionally targeted form of MBE, what is needed instead is a cognitive enhancement (Harris 2011, p. 110). Second, he expresses concerns regarding the removal of the ability to act unethically, saying that “[w]ithout the freedom to fall, good cannot be a choice; and freedom disappears and along with it virtue. There is no virtue in doing what you must” (Harris 2011, p. 104). According to Harris, a MBE which prevents someone from acting unethically eliminates any possibility of acting ethically as to do so requires a choice; acting ethically cannot be as simple as falling off a log. Harris’ third concern focuses on the potential for MBE to have the opposite effect which it intends, leading not to an increase of ethical behaviour but rather to a moral decline. He writes that “the sorts of traits or dispositions that seem to lead to wickedness or immorality are also the very same ones required not only for virtue but for any sort of moral life at all” (Harris 2011, p. 104). As such, it can be assumed from this that the prospect of a compulsory programme of enhancement, as envisioned by Persson and Savulescu, would be something which Harris would oppose.

Rakić (2013), another vocal critic of MBE, considers not whether compulsory MBE would deprive individuals of their capacity for free decision making on an individual level but from a top-down, existential societal view. He argues that by making MBE compulsory, the state would not just encroach upon the fundamental freedoms of its citizens, but also potentially deprive them of their collective capacity for morality itself. In turn, this would deny everyone a vital aspect of human existence and be itself a form of UH. He concludes that:

We need to make a choice between preserving freedom as an essential marker of our distinctively human existence and obtaining additional assurances that humanity will survive by making ME [Moral Enhancement] obligatory. If we opt for the former, we will safeguard an essential component of our human status. If we opt for the latter, we might possibly feel more confident that humanity will survive, but we do so only at the cost of giving up on a key element of our specifically human existence (Rakić 2013, p. 5).

For Rakić, implementing a programme of compulsory MBE is committing an act of UH, one that, while possibly safeguarding against the dangers of warfare or cataclysmic climate change, causes untold existential harm. As such, while he concedes that the development of MBE may indeed be necessary to prevent UH, its use should not be compulsory. Instead, he argues that it should be employed, in a voluntary capacity, in tandem with ‘classical’ compulsory moral enhancement measures, such as education and socialisation; that is, people should be encouraged to take MBE, but not coerced.^6^

Persson and Savulescu responded to Rakić’s position and criticised the idea that MBE is an UH because it infringes on one’s ability to make free and autonomous decisions (Persson and Savulescu 2013). They argue that the concept of unlimited free will, in which an agent can act in a manner not determined by the laws of physics and biology, is something which only an ultimate being has. This is something which our finite, mortal existence does not afford us. As such, MBE does not pose an unprecedented threat to our unrestricted free will as this is something which we have never possessed. MBE is simply another causal factor which influences our behaviour, just in a singularly ethical way. They also note that not all restrictions to our freedom are negative, giving the examples of disgust at eating faeces and an aversion to hurting or harming others. From here, they argue that “some non-voluntary restrictions to our freedom are beneficial” (Persson and Savulescu 2013, p. 2).

Rakić responds to these criticisms, and furthers his arguments, in his 2017 paper. He criticises Persson and Savulescu’s concept of freedom of will as a matter of degree and instead argues that it is a threshold concept, one which compulsory MBE endangers. He concludes that “compulsory moral bioenhancement deprives humans not of a degree of their free will, but of their (experience of) free will in general. As free will is a key component of our humanity, compulsory moral bioenhancement lowers our moral status” (Rakić 2017, p. 392). The issue of freedom, then, is a central sticking point in the MBE debate and one which acts as a foundation upon the ethical permissibility of compulsory and voluntary MBE.

A balance, therefore, needs to be struck between the need for compulsory MBE (as a means for ensuring sufficient uptake) to prevent UH, and the conceptualisation of mandatory MBE as, in fact, itself a form of UH. A compromise should be sought that can provide a programme of MBE that is both voluntary to avoid the risk of existential UH and state-sanctioned moral paternalism, but simultaneously sufficient enough to reduce the risk of UH via altering the behaviour of those who are most likely to put humanity, and the planet, at risk. Ideally then, what is needed is a model of MBE that would be as effective as a compulsory programme as envisioned by Persson and Savulescu, without itself needing to be mandatory; a model which allows for the ‘positive’ altering of behaviours, even in those individuals who have not undergone MBE. This leads us to epidemiological modelling, which this paper suggests can provide a viable solution to this problem.

## Epidemiology outside health

Epidemiology is the “study of how often diseases occur in different groups of people and why. Epidemiological information is used to plan and evaluate strategies to prevent illness and as a guide to the management of patients in whom disease has already developed” (The BMJ 2018). Epidemiology is the top-down study of how a subject moves through and replicates within a population, and how steps can be taken to disrupt this movement with the eventual goal of elimination. Elimination is achieved through the disruption of the infection chain. One of the most well-known methods in which this is achieved is through the tactical deployment of vaccinations. However, while it is commonly associated with the study of pathological contagions concerning health-states, its models and methodologies have been taken up in other disciplines to achieve similar, albeit fundamentally different, results.

The mechanisms that underpin the theories and observations in epidemiology have been employed as an analogy for understanding trends in a variety of complexly structured sectors, including communication (Eubank et al. 2008), criminology (Akers and Lanier 2009), linguistics (Enfield 2013), fanatic behaviours (Castillo-Chavez and Song 2003), terrorist ideology (Lafferty et al. 2008), and finance (Peckham 2014; Moellendorf 2020). The reason that epidemiology has proven useful as an analogy in the generation of insight and theories in these sectors is due to the similarities they share; specifically regarding the identification, transmission, and management of risk and the behaviour of ‘contagions’ in complexly dynamic organisations.

As just one prominent example, economists drew on epidemiological literature regarding emerging infections to understand the transmission channels of the 1997 Asian financial crisis, and how smaller players in the global financial market were able to produce systemic fluctuations to the local and global economy. The epidemiologically influenced economic models created enabled economists to identify pathways through which shock could travel between previously assumed minimally connected markets. Commentators noted that “informed by epidemiological research, the focus turned to indicators of exposure and the relationship between exposure to ‘infection’. The goal of financial ‘contagion’ theory was ultimately the ‘elaboration of causes’ to explain ‘patterns of disease occurrence’” (Peckham 2014, p. 14). The inclusion of epidemiological principles in the understanding of financial markets not only provided clarity to misunderstood economic patterns but also brought to light previously overlooked influential factors, and was so successful that the term ‘contagion’ has since become part of the standard language used by international economists and policymakers (Claessens and Forbes 2004).

Epidemiology has proven to be a useful prism through which insights can be gleaned regarding the spread of ‘contagions’ through similar complex systems. This paper proposes that similar to its use in the fields of finance, communications, and criminology, epidemiology can shed light on how a MBE could be capable of behavioural influence, without the need for such a measure to be compulsory, as proposed by Persson and Savulescu. To argue this point, however, a brief explanation of the dynamics of transmission and process of vaccination is needed.

## Herd immunity and infection chain disruption

Vaccinations work by introducing either a weakened or dead version of a biological entity to an individual’s immune system. This introduction prompts that system to produce antibodies which, after the initial infection has been cleared from the body, remain to a degree within the blood. It is this continued presence which facilitates an active immunity and means that the individual is potentially less likely to become re-infected, or in some cases, less likely to suffer from infection-related complications. The immune system is enabled to react faster in cases of subsequent infection. This is known as the direct effect of vaccination (Halloran et al. 1991).

Vaccinations also have indirect effects, one of which is herd immunity.^7^ For a contagion, such as a biological pathogen, to spread throughout a population, three things are required. First is the agent, this is the contagion which will spread throughout any given population, such as the flu or measles. Second is a host, the individual who will incubate the agent and who is typically considered as being ill. Third, is a habitable environment an agent can utilise to travel to a new host. By eliminating one of these triadic factors, a contagion is unable to spread through a population and as such, over time and without subsequent infection reintroduction, the contagion can be eradicated. If enough of a population undergo immunisation against a contagion, the chance of any individual host encountering a vulnerable person is reduced, which in turn decreases the spread of the contagion. Eventually, this reduction occurs to such a degree that the contagion is unable to spread so fast that a population cannot recover from it; what is commonly known now, thanks to the COVID-19 pandemic, as having an *r* value less than one. Therefore, the prevalence of contagion eventually falls. This fall, in conjunction with other forms of intervention, can eventually lead to the complete eradication of contagion from a population, and in the most successful cases, its complete wild-extinction, as with Smallpox in the 1980s (Fenner 1988).

Smith identified that for this herd immunity to take place a certain proportion of a population must undergo immunisation (Smith 1970). The minimum number of individuals that need to undergo vaccination in order for herd immunity to take effect is known as the ‘herd immunity threshold’ (Mallory et al. 2018). Below this threshold, a contagion is likely to propagate, above the threshold, it is unlikely to propagate successfully. The threshold differs according to environmental, agent, and host factors. Therefore, for an accurate threshold for any particular contagion to be identified, these individualised factors must be carefully considered. Herd immunity is important because it protects the vulnerable subpopulation of individuals who have not undergone immunisation themselves, for whatever reason. These individuals become insulated from infection by a contagion when herd immunity is established, and thus they can gain from the benefits of immunisation without actually having to be immunised themselves.

This paper will demonstrate how this phenomenon of herd immunity can be utilised in the discussion regarding MBE. Specifically, it posits that if enough of a population were to undergo MBE and pass what would be the equivalent of the MBE threshold, then the prevalence of morally undesirable behaviour could decrease. Such a moralising effect of MBE would alter not only the actions of those who undergo MBE but also those who have not as a result of its indirect effect. Such an argument, however, depends on establishing that there is a similarity between (un)desirable behaviour/morality and the spread of biological contagions. Such a comparison has been well explored in the literature on social contagions.

## Social contagions

The view that ideas, behaviours, concepts, and specifically morality, spread through populations in an analogous manner to that of a contagion is nothing new. In 186 B.C., the Roman historian Livy wrote of the Bacchanalia orgies that “[t]his pernicious scourge made its way from Etruria to Rome like a spreading infection…” (Livy 2018, p. 231). The comparison between the spread of behaviours and that of disease continued from this point, through the various ‘dancing plagues’ of the middle ages (Miller 2017), becoming notably popular around the end of the nineteenth century, prominently featuring in the works of Baldwin (1894), le Bon ([1895] 2001), and de Tarde (1903). Each of whom was interested in the behaviour of crowds and of ‘group suggestion’.

The theory of behaviour as contagious has more recently been employed to understand and model specific patterns of behaviours in individual organisations and groups, as illustrated in the work of Gladwell (2001), Sooknanan and Comissiong (2017), House (2011), Robinson and O’Leary-Kelly (1998), Connolly and Åberg (1993), Bettencourt et al. (2006), as well as Ambrose et al. (2013). Each argued that rather than considering the behaviour of individuals on a micro-level, individual behaviours could be understood on a macro-level, with their prevalence and movement traced according to the same mechanisms at play in epidemiology. Such an approach can furnish researchers and policymakers with the behavioural information required to make informed decisions regarding the management of groups when data is scant or when theories need verification.

Envisioning behaviours, according to these macro-level models, can enable interested parties to predict the movement of undesirable behaviours and potentially implement measures to restrict the spread of radical and destructive ideologies. This includes phenomena as severe as fanatic behaviours (Castillo-Chavez and Song 2003), terrorist ideology (Lafferty et al. 2008), as well as violent crime and burglary (Ormerod et al. 2001). Such an approach, regarding the threat of terrorism, has even been acknowledged by the US Department of Homeland Security, according to whom “the capabilities and laws we rely upon to defend America against terrorism are closely linked to those which we rely upon to deal with non-terrorist phenomena such as crime, *natural disease*, natural disasters, and national security incidents” (Office of Homeland Security 2002, p. 4). Additionally, with the advent of social media and the unprecedented rate at which messages, ideologies, and opinions are now communicated, this field is receiving increasing interest with more complex behavioural contagion models being developed to attempt to account for this technological method of transmission (Piedrahita et al. 2018; Sprague and House 2017).

In their study observing the contagiousness of rudeness within organisations, Foulk et al. (2016) noted that the transmission of undesirable behaviours is not just limited to the interaction between the original ‘infected’ individual and those with whom they come into contact. Third parties with whom the second-generation infected individual interacts with are just as at risk of becoming behaviourally infected, even though they never interact with the negative behavioural progenitor. Consequently:

…like a true virus, the effects of even a single act of rudeness may manifest themselves in multiple parties within the organization. Accordingly, we specifically hypothesize that the rudeness of the first party will have indirect affective, cognitive, and behavioral effects for third parties (Foulk et al. 2016, p. 53).

A pattern of rude behaviour can propagate itself through the replication of its behaviours within an individual, which are then passed onto another via direct or indirect contact. Crucially, this process is not restricted to a single generation but can potentially continue ad infinitum, provided that susceptible individuals are exposed to the behaviour within an environment which allows for its transmission. This is made all the more possible given that “like the common cold, the types of negative behaviours that can be contagious are everywhere” (Foulk et al. 2016, p. 50). Indeed, unlike many biological contagions that require exact conditions under which to spread, as specialised vectors can only infect a particular type of host, behaviours can infect anyone, at any time, and are dispersible across a substantial range.

Furthermore, according to multiple studies, much like the broader breadth of behaviour, both desirable, as well as undesirable moral attitudes and behaviours, are also communicable (Huddart and Qu 2013; Eskine et al. 2013; Hofmann et al. 2014). Concordantly, there is a case to be made, or a hypothesis to be put forward, that a significant, influential factor in the creation of an instance of immoral behaviour is a preceding case of immoral behaviour; one begets the other.

However, as with the transmission of biological contagions, if the chain of replication of negative behaviour is interrupted, then that behaviour would cease to be passed on and eventually it would be subsumed by the desirable contagious behaviours, which work according to the same principle of transmission and replication (Bass et al. 1987), provided that no new instances of rude behaviours were introduced to the population. For example, if one was to introduce a rudeness inhibitor which would radically decrease the disposition of individuals to be rude, at a level high enough to exceed what this paper will term ‘the behavioural immunity threshold’, not only would this work towards preventing instances of new rudeness occurring (the direct effect), but also interrupt the chain of transmission of rude behaviours by dramatically reducing the chance of a vulnerable individual from coming into contact with such behaviours (the indirect effect). Consequentially, even those individuals who have not undergone rudeness inhibition would, to a degree, be insulated against the contagious rudeness of others and as such benefit from the herd immunity of the organisational enhancement.

It is along this line of thinking that this paper wishes to move, but rather than discussing the contagiousness of low-intensity negative behaviours like rudeness, it will consider how MBE can interrupt the transmission chain of ‘ultra-high-intensity’ negative behaviours, such as those which can potentially lead to UH, and as such be effective without the need to be compulsory.

## MBE and the herd immunity of morality

The understanding of MBE as employed by Persson and Savulescu acknowledges the direct effect of the enhancement, but not its indirect effect. According to their interpretation, only individuals who undergo the enhancement are those who will reap any benefit regarding an alteration to their moral faculties. These individuals would no longer ‘generate’ behaviours likely to bring about UH, and as such, due to this process being, in essence, insular, every individual would need to undergo it in order to eradicate unethical behaviours from the target population; be that a city, country, or more likely given the scale of UH, the planet. To leave a single individual unenhanced would allow for the endurance of the risk of UH, making the efforts of those who have undergone MBE redundant as this small group could undo the morally beneficial behaviours of the larger populace. However, this interpretation ignores the potential indirect effect of MBE in two key ways: the interruption of the transmission of morally undesirable behaviours, and the contagious quality of behaviours that are morally desirable.

First, if a large enough proportion of the target populace underwent MBE so as to reach the behavioural herd immunity threshold, then the transmission of undesirable behaviours would begin to decline. This is due to the removal of the susceptible host from the transmission triad, as discussed earlier. The difference now is that in the previous triad the entity being transmitted was a biological agent, whereas now it is a form of behaviour. Those people who perpetuate undesirable behaviours would encounter the morally unenhanced in smaller and smaller numbers, and as such, the chances for them to spread their behaviours would also decrease. This decrease would have a ripple effect, reducing the instances of systematically generated undesirable behaviours not only in the first instance but also any subsequent ‘infections’. As such, the chances of any singular individual coming into contact with a person from whom they could catch a negative behavioural contagion would decrease over time. This conceptualisation is similar to how some radical and violent crimes have been mapped, and measures planned, with the goal of curtailing their spread (Sooknanan et al. 2013).

By disrupting the chain of behavioural infection, one can steadily reduce the prevalence of undesirable behaviours until those behaviours only occur spontaneously. At this point, more contemporary forms of moral enhancement, such as education and social interaction, can come into play, tackling the source of these negative behaviours. This parallels how, in cases where immunisation has not been as effective as desired, and an individual becomes ill with a biological agent, other medical services are offered to assist in their recovery and prevent the spread of that pathogen.

This, in turn, relates to the potential for UH which small groups or individuals can pose. Namely, that even with the effect of a behavioural herd immunity taking hold, insulating the unenhanced against behaviours that could lead to UH, this does not categorically prevent such an UH from coming about. Small groups or individuals seeking to cause deliberately UH are not directly prevented from doing so by the effect of behavioural herd immunity. However, this paper is not suggesting that such an effect is, in-and-of-itself, sufficient to prevent such an outcome. Other social measures and institutions—such as the police, counterterrorism, environmental agencies, amongst others—will still need to exist as a means of tackling such sporadic instances of UH causing behaviours. What this paper is suggesting is that the instances and spread of such behaviours could be significantly reduced through a voluntary programme of MBE to the same degree as would be provided by a compulsory programme. This would then allow for contemporary measures and institutions to target their time and resources on the instances and individuals who remain a threat.

Second, the decrease in the transmission of immoral behaviours is not the only indirect effect that this paper postulates MBE could have. The enhancement could also increase the instances of people behaving in morally desirable ways and crucially, these morally desirable behaviours are subject to the same transmission phenomena as their negative counterparts (Mayer et al. 2009). One can ‘catch’ behaviour that would increase the likelihood of ‘ultimate good’,^8^ just as one can ‘catch’ those behaviours which lead to UH. As such, at the same time that those individuals who have not undergone MBE would become less likely to interact with someone from whom they would catch a negative behavioural contagion, they would also be immersed in an environment saturated with people acting in a morally positive way due to their undergoing MBE. This means that those individuals would be at a high ‘risk’ of contracting beneficial behavioural traits, regardless of whether they have been enhanced, due to the sheer number of individuals with whom they would come into contact. This would then reinforce and maintain positive behavioural traits because the unenhanced would constantly be ‘re-infected’ with the desirable behavioural contagions.

In a population in which the vast majority of individuals have undergone MBE then, it is not beyond the realm of possibility that the population would be protected from a self-inflicted UH as a result of the disparity between their moral and cognitive faculties. If behaviours act like contagions, which this paper has suggested they do, then it is possible that other epidemiological phenomena can be observed in the formation and spread of behaviour, such as herd immunity. Consequently, while it would indeed be necessary for a large proportion of individuals to undergo MBE in order for the collective effect of herd immunity to take hold, and there is room for discussion about how this might be achieved voluntarily, this is significantly different from arguing that such an intervention would need to be compulsory to be effective.

## Conclusion

The phenomenon of herd immunity is one that is critical in the field of vaccine epidemiology and public health. Once it takes effect, even those individuals who are unable to undergo vaccination are still able to benefit from a functional immunity from a biological agent. As such, a compulsory and universal programme of vaccination is not always necessary to achieve a sufficient protection rate against a contagious biological agent. It is this same line of reasoning which this paper has sought to employ, envisioning MBE as a form of vaccination against those types of behaviour that would lead to the realisation of UH. Consequentially, this allows for the possibility of sufficient protection against the undesirable behaviours that would lead to UH without a need for a universal and compulsory enhancement programme.

This stands in contrast to the claim made by Persson and Savulescu that in order for MBE to be meaningfully effective at eliminating UH it would need to be compulsory (Persson and Savulescu 2008, p. 174). This paper has argued that a voluntary programme of MBE could be effective at reducing or eliminating the risk of UH provided that a large enough portion of a population undergoes the measure. This would not only disrupt the transmission chain of undesirable behaviours but also provide an environment in which the morally unenhanced are likely to ‘catch’ morally positive behaviours, decreasing the chance of UH while increasing the likelihood of an ‘ultimate good’.

This paper recognises that the number of individuals who would need to undergo MBE in order to achieve this effect would need to be significant to meet the enhancement threshold. Further research would need to be undertaken in order to identify where this threshold is, and even then, it may be impossible to identify due to the complexity of human behaviours, the diversity of environments in which people live, and the multitude of ways in which individuals and groups interact. However, the difficulty of identifying this point is not the same as saying that such a threshold does not exist, nor that the only way to ensure humanity’s survival is to enforce a programme of compulsory MBE. There remains the possibility that such a threshold is so high as to require compulsory MBE as the only means to achieve it. However, this is not a given, and until it is demonstrated that compulsory MBE is the only method for averting the threat of UH, then it would seem premature to argue in its favour. This is especially true if the same effect sought by Persson and Savulescu can be realised via less coercive means.

The question also remains regarding why anyone would come forward to undergo voluntary MBE, let alone a large enough proportion of the population to bring into effect a behavioural herd-immunity. After all, governments globally increasingly struggle to maintain vaccination rates. A potential motivator could be a sense of duty and social responsibility; that one should do their part not only to further their ends but because they have a responsibility to act morally for the good of others. Rakić postulates that there could be a motivation derived from a self-interested desire to be happy, arguing that the more moral one is, the happier they tend to be, and vice versa. As such, if one wants to increase the likelihood of being happy, then it would arguably be in their interests to volunteer for MBE (Rakić 2018). Given the recent concerns from some of the global populace regarding a COVID-19 vaccination, Rakić’s suggestion appears less than convincing.

More work on the comparison between MBE, vaccinations, and behavioural contagions is needed before a definitive answer to the question of whether MBE should(n’t) be compulsory is found. However, with a low likelihood of a universal compulsory programme of MBE being enacted for a variety of political, ethical, and social reasons, any way to achieve the security from UH without enforced enhancement is likely to be crucial.

## Footnotes

1 I feel it would be prudent to make the reader aware that this paper was written before various governments began floating the idea that targeted herd immunity could be a possible long-term method to tackle the COVID-19 pandemic. This is something which I fervently oppose as this is most often articulated in terms of sacrificing the most vulnerable in society to secure not only people’s lives but also economies. While I believe such methods are outright monstrous in terms of tackling the COVID-19 pandemic, I still believe such modelling can be of value in the voluntary vs compulsory MBE debate.

2 It should be noted that while this article makes the following assumptions, that is not to say that this article necessarily agrees with such assumptions, or that alternative views or conclusions can or should be disregarded. This paper is merely utilising these conclusions as a means of better framing the debate with which it is primarily concerned.

3 This article takes traditional MBE to be those forms of societal practice which aim, via explicit instruction or passive consciousness-raising practices, to improve the moral behaviours and ethical understanding of individuals (e.g. education, socialisation, etc.).

4 UH, as a founding rationale for MBE, be it voluntary or compulsory, has been criticised, most notably by Wiseman (2016). While he concedes that the world, as it is currently, is facing significant and potentially cataclysmic issues, this does not, in turn, legitimise the use of MBE.

5 It should be noted that in the same issue of the *Journal of Applied Philosophy*, another significant paper on MBE was published, this one by Douglas. His paper, entitled *Moral Enhancement*, presented an argument in favour of MBE. However, it does so via a more moderate degree than that of Persson and Savulescu, omitting suggestions to the compulsory and universal application of MBE.

6 Where exactly the line is between promotion and coercion is a sticky point of contention and one which Rakić does not elaborate on in any great detail.

7 Another term for this effect is ‘herd effect’. However, for the purposes of this paper, the term herd immunity will be employed.

8 Ultimate good, in the context of this paper, is taken to be merely the opposite of UH. Whether that entails a continuation of the *status quo*, or a refutation in favour of a state of existence even more beneficial, is something which this paper does not intend to explore.
